# Sailing to and Docking at the Immune Synapse: Role of Tubulin Dynamics and Molecular Motors

**DOI:** 10.3389/fimmu.2018.01174

**Published:** 2018-05-30

**Authors:** Noa Beatriz Martín-Cófreces, Francisco Sánchez-Madrid

**Affiliations:** ^1^Servicio de Inmunología, Hospital Universitario de la Princesa, Universidad Autónoma de Madrid, Instituto de Investigación Sanitaria Princesa (IP), Madrid, Spain; ^2^Centro de Investigación Biomédica en Red de Enfermedades Cardiovasculares (CIBERCV), Madrid, Spain

**Keywords:** immune synapse, cytoskeleton, T cell receptor, centrosome, dynein, dynactin, microtubule, molecular motor

## Abstract

The different cytoskeleton systems and their connecting molecular motors move vesicles and intracellular organelles to shape cells. Polarized cells with specialized functions display an exquisite spatio-temporal regulation of both cytoskeletal and organelle arrangements that support their specific tasks. In particular, T cells rapidly change their shape and cellular function through the establishment of cell surface and intracellular polarity in response to a variety of cues. This review focuses on the contribution of the microtubule-based dynein/dynactin motor complex, the tubulin and actin cytoskeletons, and different organelles to the formation of the antigen-driven immune synapse.

## Introduction

The immune synapse (IS) is a highly organized structure at the interface between a T cell and an antigen-presenting cell (APC) that is initiated by antigen recognition through the T cell receptor (TCR) and supported by the complex network of cell skeletons (1–3). In particular, the role of tubulin- and actin-based skeletons has been studied on the polarization of intracellular organelles at the IS and the organization of specific adhesion molecules and signaling receptors at the plasma membrane (4–6). Candidates to regulate intracellular traffic and cell organization are the tubulin-based dynein/dynactin molecular motors (7, 8). Various strategies have been used to study this issue, e.g., omics-based techniques (proteomics and lipidomics) upon biochemical extraction and imaging of live or fixed cells through fluorescence and/or electron microscopy. Major issues include determining the molecules that perform their function at the T cell surface, during T cell activation, the components delivered to the cell surface at/or near the IS that sustain or switch off T cell activation and the relevant mechanisms that control their transit to the IS upon activation. Motor protein complexes such as dynein/dynactin lie at the core of these issues. They regulate the movement and positioning of different cellular components, and generate internal forces.

Many studies support the principle of action and reaction processes during IS organization. The scanning of the APC by the T cell through initial adhesive contacts based mostly on lymphoctye function-associated antigen-1 (LFA-1), and the actin reorganization in the T cell impact on the ability of APCs, such as dendritic cells (DCs), to mobilize their intercellular adhesive molecules (ICAM-1 and -3) and subsequently the major histocopatibility complex class II molecules (MHC-II) (9). Recently, LFA-1 activity on T cells has been found to be important for ICAM-1 clustering at the DC, but not for MHC-II. The co-localization of MHC-II and ICAM-1 is mainly abrogated by drugs disrupting the actin cytoskeleton, which reduce MHC-II mobility while increasing ICAM-1 mobility (10). Most of the filamentous actin of a T cell engaged in an IS is found at a highly motile, contractile lamella used by the T cell to interact with the APC (11). The balance between actin filament polymerization and depolymerization establishes a retrograde flow that mediates continuous movement (12). Actin polymerization in filaments at the cell edge and depolymerization near the IS center directs the movement of different surface proteins, such as the TCR/CD3 complex (13). The organization of fluctuating molecules in clusters of different sizes allows the scaffolding of signaling networks that are highly efficient for the transmission of external cues to the intracellular milieu (14–16). All these processes can influence the APC. Forces usually come in pairs (action–reaction). Therefore, since protein-based complexes change their speed upon cell–cell contact, there must be an acceleration, which will depend on the net force applied and the mass of the object. This makes the actions exerted by T cell–APC contacts relevant for changes occurring in both cells. The mass of the objects (protein complexes) involved may change, as well as their ability to interact with large elements (cytoskeleton) that confers a kind of resistance and their capacity to undergo intramolecular changes that make the molecule itself different in terms of intramolecular stiffness or rigidity. All these events will affect the acceleration of the objects. In this regard, a conjugated T cell might not be considered as a “rigid body,” since it is highly plastic and its components change their position and shape, deforming the overall object (T cell). These circumstances confer relevance to every single event on receptors, cytoskeleton, and organelle dynamics during IS formation, therefore pointing to molecular motors.

The IS also behaves as an “active zone” acting as a platform for localized vesicular trafficking (17). This region corresponds to a low-actin area, which allows the microtubule (MT)-mediated transport of endosomes and vesicles toward the pericentrosomal region, near the IS and from there to the plasma membrane (18, 19). Although internalization of TCR/CD3 may occur randomly at any part of the cell surface, recycling is mainly focused to the T cell–APC contact area, leading to the polarized accumulation of this receptor at the IS (20). The effective membrane traffic is a relevant, quantifiable process, both in resting and activated T cells, for the balance of the cellular localization of very different components, from the TCR to integrins and signaling components, such as kinases and adaptor proteins. It is also important to measure cell degranulation and secretion, as well as to evaluate the compartments dedicated to degradation, such as lysosomal-dependent autophagic or endosomal partitions. Hence, the contribution of lateral membrane motility to the recruitment of TCR/CD3 at the IS is facilitated by an intracellular pool of the complex associated with recycling endosomes to balance metabolic steady state (4). These endosomes are stores of signaling molecules and adaptor proteins and play a role in delivering them to the plasma membrane at the IS (21). The regulation of these internal movements depends on different molecular motors. In this review, we offer a perspective on the molecular players and mechanisms that may be contributing to the internal forces that control organelle positioning and function at the T cell–APC contact area; in particular, dynein/dynactin complexes.

## Coordination of Actin and Tubulin Cytoskeletons at the T-APC Contact Area

**Figure 1:**
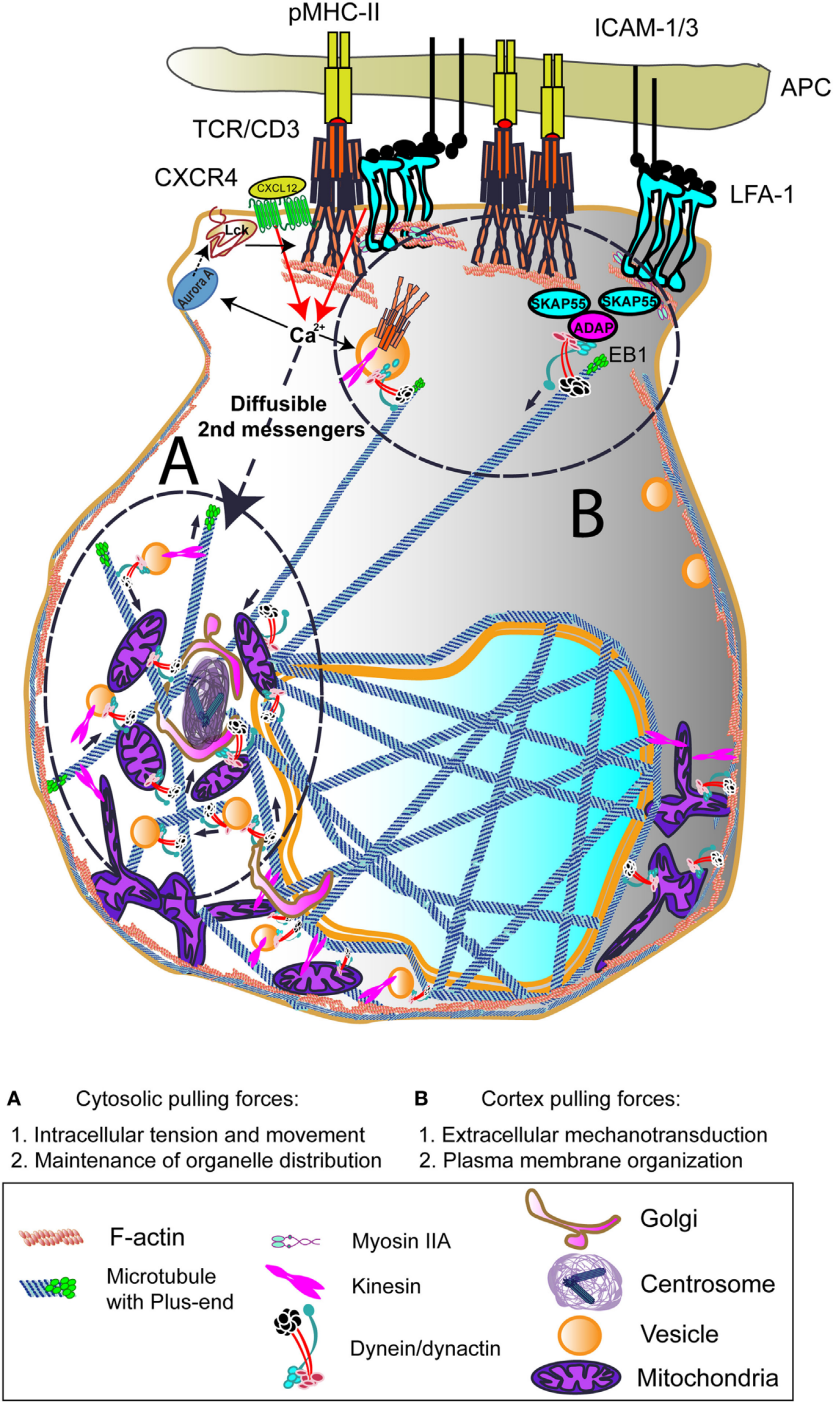
Dynein-driven forces acting on centrosome polarization during early T cell activation. Centrosome polarization upon T cell receptor (TCR) activation can be mediated by cortex and/or cytosolic pulling forces. TCR, lymphoctye function-associated antigen-1 (LFA-1), and CXCR4 binding to its ligands involve rapid increases in Ca^2+^ intracellular influx. This can activate Aurora A, which will favor leukocyte C-terminal Src kinase (Lck) activation and subsequent CD3 ITAMs phosphorylation at the CD3/TCR complex. The activation of the centrosome and associated molecules is probably due to diffusible secondary messengers such as the Ca^2+^. The interaction of dynein/dynactin complexes and other motors with intracellular organelles and the cytoskeleton may induce the force needed to move the centrosome toward the immune synapse (IS). At the IS, the interaction with TCR/CD3/SKAP55/ADAP or LFA-1/SKAP55/ADAP may serve to dock growing microtubules (MTs) and to pull the centrosome to the IS. End-binding 1 (EB1) may interact directly to CD3ζ subunit of the CD3/TCR complex. The growth and shrinkage of MTs at this zone would also create pulling forces. The images in the figure are not scaled.

**Figure 2:**
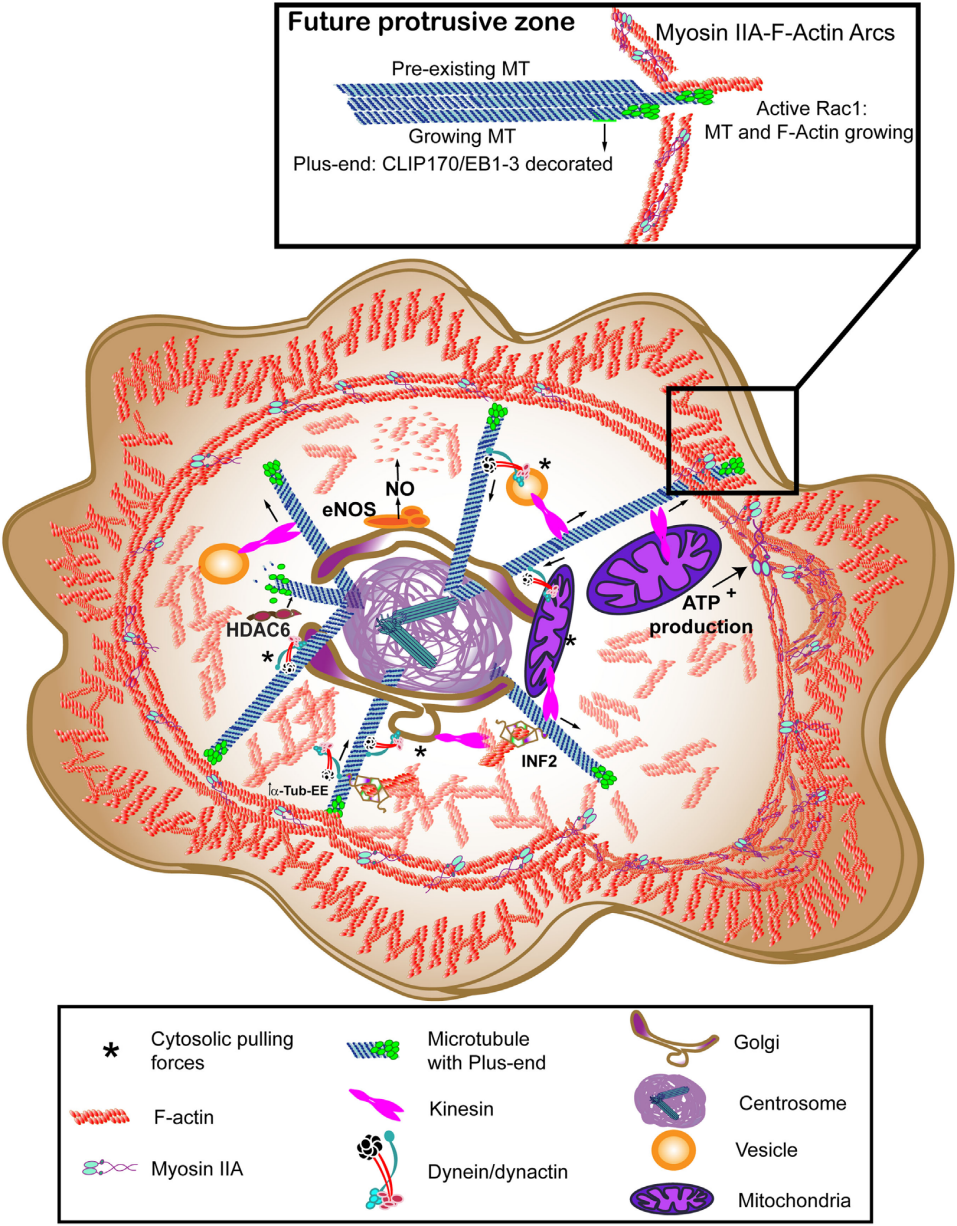
Molecular motors at motion to rearrange the cytoskeleton at the immune synapse (IS). Myosin IIA provides the lymphoctye function-associated antigen-1 (LFA-1)-dependent actin ring with contractile activity, thereby helping the centripetal movement of surface proteins. Dynein/dynactin may interact directly with receptors or move vesicles to allow recycling, walking toward the minus-end of microtubules (MTs) (centrosome). Kinesin-1 helps the traffic from the centrosome to the periphery. Vesicular traffic allows secretion and mitochondria can provide the adenosine tri-phosphate (ATP) needed for Myosin IIA activation. The forces exerted by these motors between the organelles and the cytoskeleton constitute the cytosolic pulling forces, that may provide a docking mechanism for the centrosome. The translocated centrosome provides the IS with multiple signaling, scaffold and modifying proteins that can regulate relevant post-translational modifications (PTMs) for actin or tubulin cytoskeletons, such as endothelial nitric oxide synthase (eNOS) for β-actin nitrosylation, histone deacetylase 6 (HDAC6) for deacetylation, or inverted formin-2 (INF2) to allow detyrosination of MTs. The images in the figure are not scaled.

The interplay of actin and MT skeletons with surface receptor complexes coordinates the forces applied on the T cell and those exerted by the T cell (Figure 1). A specific correlation between MT and actin areas has been largely analyzed in different cellular systems. It is clear that MTs and actin territories partially overlap. The activation of Rac1 regulates both actin polymerization and MT growth at the leading edge during migration (22). At the IS, MTs growing from the translocated centrosome (18) may benefit from TCR and integrin-mediated activation of Rac1, paralleling the retrograde flux of actin (Figure 2). Activated Rac1 collaborates with Rab11 and FIP3 at endosomes to control actin dynamics and tracking forces at the IS (23) and endosomal clathrin coordinates actin polymerization at the same location, thereby controlling T cell activation (24). Since endosomes re-localize near the IS thanks to the centrosomal positioning, it is conceivable that MT skeleton collaborates with actin for sustained tracking forces. This may be done through changes in the MT network by polymerization and depolymerization at plus ends of MTs during centrosomal repositioning and once it is located at the IS (18, 25, 26). However, disruption of Aurora A activity or expression, which reduces MT growing from the polarized centrosome (but not its polarization) and vesicular traffic at the IS, does not prevent actin dynamics in CD4 T cells. Aurora A activates leukocyte C-terminal Src kinase (Lck), a tyrosine-kinase involved in early TCR/CD3 phosphorylation at the plasma membrane, but the blockade of Aurora A does not affect Lck activity enough to prevent the docking of the centrosome in CD4 T cells (27). Lck activity on CD3 ITAMs is required for correct centrosome polarization (28). This cascade of kinases might be rapidly activated by Ca^2+^ influx, since Ca^2+^ promotes immediate Aurora A activation (29). It has been postulated that CD8 T cells require Fyn tyrosine-kinase for centrosome movement and Lck for docking (30). Previous work showed that Fyn is relevant for centrosomal polarization mainly in the absence of Lck. Full localization of centrosome at the IS is prevented in CD8 Fyn-deficient T cells when anti-CD3ε-coated beads are used. Moreover, Fyn-deficient OTI CD8 cells show maximal inhibition of centrosomal polarization under low stimulation conditions, such as partial agonist and antagonist peptides, but only mild effect when activated with agonist peptide (31). Thereby, other elements involved in the activation of the T cell, such as LFA-1 integrin and MHC-TCR interaction may direct the complete stimulation of centrosomal polarization.

A dual action of the centrosome on actin dynamics may exist. On the one hand, it can provide positive regulators from the associated Golgi apparatus and secretory machinery to increase cell–cell adhesion. On the other hand, negative regulators of actin polymerization such as nitric oxide produced by endothelial nitric oxide synthase (eNOS) (32) can help the fine-tuning of actin dynamics to prepare the clearance of actin structures at the center of the contact for secretion. The final recovery of actin at the IS and the switch off of the activated T cell can be achieved through the delivery of negative regulators, such as cytotoxic T-lymphocyte-associated antigen 4, to the IS from the endosomal associated systems (33). The organization of the polar retrograde flux of actin in the geometrical shape generated at the interface between the T cell and the APC generates a low-viscosity “sink” for inward flow of signaling microcluster in the T cell (34). There, actin regulators probably cluster, such as cofilin, profilin, and coronin. Golgi-resident eNOS coordinates centrosomal positioning at the IS with actin dynamics by decreasing the actin retrograde flux through modification of the actin binding to profilin by β-actin nitrosylation (32). Profilin is a major actin-binding protein in different cells (35), which makes it a predominant substrate for actin polymerization. Recently, two independent studies have demonstrated its collaboration with Ena/VASP complex and with formins to organize actin polymerization rather than Arp2/3 complex. Through cooperation with profilin, actin increases its ratio of incorporation to formin-bound filaments and helps Ena/VASP complex to elongate the distal lamellipodia (36, 37). The ability of different formins to increase actin polymerization can also help the initiation of finger-like protrusions at the plasma membrane in coordination with lamellipodia extension. FMLN3 formin, in cooperation with mammal diaphanous-related-formin (mDia)2, favors this process; however, this is not the case for FMLN2 (38). FMLN1 and mDia1 targeting showed no effect on filopodia formation and actin accumulation during T cell interaction with APC. The knockdown of either Arp2 or Arp3 converted the lamellipodia-based scanning of the APC into a filopodia-based interaction (39). It has been described that the formation of Arp2/3-dependent F-actin foci at TCR microclusters at the IS may facilitate the formation of protrusions toward the APC (40). Filopodia—also referred as microvilli or microspikes—seem relevant to the initial scan of the APC by the T cell through a tyrosine kinase- and actin-independent TCR–pMHC interaction (41). Therefore, the actin organization upon activation relies largely in Arp2/3 and formin activities. Recently, FRAP analysis of the cell cortex of T cells has determined the presence of two F-actin subsets: formin-nucleated, long filaments of about 500 nm showing long turnover times and Arp2/3-nucleated, short filaments of 50 nm with fast turnover times and actin free barbed ends. Also, Arp2/3 activity was more prominent than formin upon TCR activation, but the formin activity endorsed longer filaments on the external lamellipodia. The use of both super-resolution stimulated emission depletion (STED) microscopy and lattice light-sheet microscopy (LLSM), allowed the identification of a more internal network of actin at the IS, different from the lamellipodia and complementary to it, that may help the intracellular traffic (42). In this regard, profilin ability to bind formins can help the interconnection between F-actin and MTs, as formins can bind simultaneously both elements through their formin homology (FH)1 and FH2 domains (43), which may be also a relevant mechanism to generate protrusions at the plasma membrane (see Figure 2).

Microtubules are polymers of α- and β-tubulin heterodimers bound in a head-to-tail manner. This organization gives rise to MT polarity, with plus- and minus-ends, depending on their rate of polymerization (44). The conventional formin mDia has been shown to bind end-binding 1 (EB1), CLIP170, and APC, a group of proteins involved in the growth of MTs at their plus-ends, or tips (43). mDia-deficient mouse presents different alterations in T cell development and activation (45). Indeed, Arp2/3 accounts for TCR recycling and cell–cell adhesion in conjugates, without affecting centrosomal positioning, whereas mDia and formin-like protein 1, two canonical formins, affect centrosomal localization. Their effect on actin is antagonistic (39). Inverted formin-2 (INF2), a non-canonical formin, regulates the centrosome translocation, but does not seem to affect actin during IS in T cells (46). At any rate, Rac1 activity seems relevant for centrosomal polarization to the IS (39, 46). Both actin exclusion and centrosome recruitment at the center of the IS, together with the vesicle and secretory machinery of the cell, allow the correct signaling and recycling of receptor microclusters and also focus secretion (6, 47, 48). In cytotoxic T-lymphocytes (CTLs), the secretion of lytic granules recruited to the polarized centrosome at the target cell area (49) is facilitated by the clearance of central cortical actin in coordination with calcium influx (50, 51). On the other hand, local clearance of actin at the site of docking and delivery of the granules has been defined in the killing immunological synapse organized by NK cells. This effect is dependent on the action of Coronin 1A, a protein able to interact with F-actin and MTs that uses Arp2/3 to destabilize F-actin (52, 53). Coronin 1A seems to be dispensable in T cells for antigen-recognition events, but not for migration (54). To fulfill their ability to engage different target cells serially, CTLs seem to recover cortical actin upon secretion, thereby stopping this process (55). The role of the centrosome in driving the localization of the lytic granules at the target cell area, and the possible role of centrosome-associated, Golgi-resident eNOS in actin clearance point to a role of tubulin skeleton in fine-tuning actin-based cytoskeleton dynamics.

## Dynein Motors Shape the IS

Experimental evidence on this issue arises from the observation of the polarization of the cytoskeleton at the IS and the associated changes of intracellular organization. In the T cell, the centrosome, together with the Golgi apparatus, secretory and recycling machinery and mitochondria, localize at the IS. An active growth of MTs from the centrosomal area organizes an MT network that helps traffic at the IS (56). An array of molecular motors is able to walk between the two ends of MTs, transporting different organelles along these trails. Also, actin-based molecular motors can act on actin structures to increase tracking forces at the IS, probably helping movement. These molecular motors are important for cell polarity and are mostly represented by dyneins, kinesins, and myosins (Figures 1 and 2).

Cytoplasmic dynein complex belongs to a large family of MT motor proteins involved in intracellular transport; it is an MT minus-end directed, motor protein complex of about 1 MDa, that comprises two heavy chains containing the AAA motor and MT binding site, two different intermediate chains, among them the p74 subunit (DIC) and several light and intermediate light chains. These chains provide interaction with a plethora of proteins in cells, conferring to the motor the ability to interact with multiple cargoes. p74 interacts with dynactin p150^Glued^ subunit, and p150^Glued^ is able to interact with MT through its first 200 amino acid residues at the N-terminus CAP-Gly (cytoskeleton-associated protein glycine rich) domain and a basic region. Dynactin complex enhances dynein processivity while regulating its localization (57, 58). Cytoplasmic dynein accumulates at the periphery of the T-APC contact and associates with ADAP (adhesion and degranulation promoting adaptor protein; SLAP130/Fyb) (59), as does dynactin (60). This interaction may generate the pulling force needed to polarize the centrosome to the IS; e.g., cortex pulling forces (Figure 1). These cortex pulling forces would be exerted from the ring of activated LFA-1 integrin, favored by ADAP upon TCR activation. Accordingly, the knockout mouse for ADAP shows deficient T cell LFA-1-mediated adhesion (61, 62). Although actin polymerization is normal in the knockout cells, the absence of ADAP is essential for T cell proliferation and adhesion. Indeed, ADAP is a scaffold protein that connects to SKAP55 and regulates its stability and half-life by preventing its degradation at the proteasome (63). SKAP55 connects with the actin cytoskeleton and its deficiency causes an effect similar to that of ADAP (64). Therefore, it is conceivable that, through connection with ADAP, cytoplasmic dynein might exert a regulatory role on interconnecting MTs and cortical actin at the IS, producing the pulling forces at the cortex. The disruption of the dynein/dynactin complex de-localized LFA-1 from the external zone of the IS, showing a scattered pattern (60). In these conditions, the centrosome was not polarized at the IS, without affecting the number of conjugates formed with APCs. Indeed, dynein has also been proposed to move TCR/CD3 complexes along MT toward the center of the IS, enhancing their motility and signal termination in mouse cells (65). Coordinated dynein/dynactin activity was also found essential for sustained T cell activation, based on centrosome polarization (60).

Cytoplasmic pulling forces are now matter of study and exemplify a mechanism *via* dynein/dynactin complexes to generate traction during intracellular transport and cell shape maintenance (Figure 1). Cytoplasmic pulling forces are based on the net forces applied by molecular motors to get together the components of organelles such as the Golgi, as well as the traction exerted on skeletons for movement. Dynein/dynactin-mediated cytosolic pulling forces may be relevant for the localization of the centrosome, given the high number of organelles and vesicles which are interconnected by MTs around it, and their proximity to the IS (24, 66). The study of large protein complexes in cells is difficult due to the high number of subunits and the ability of cells to compensate some effects when protein complexes are disturbed or the protein expression of their subunits diminished. In the case of dynein/dynactin, either the silencing of cytoplasmic dynein heavy chain 1 or a high overexpression of the p50-dynamitin-GFP subunit of dynactin in human T cells prevented the correct polarization of the centrosome. A sustained, long-term overexpression of p50-dynamitin-GFP [obtaining a ratio of more than 4:1 for p50-dynamitin:p150^Glued^ proportions in the dynactin complex (67, 68)] in Jurkat cells prevented the interaction between p74-dynein intermediate chain and p150^Glued^. This effect correlated with a dispersed localization of the TCR, as well as with a de-localized centrosomal positioning (60). A recent study shows that dynein motor, which can form different complexes in cells by changing its partners, may play a dual role in T cell activation, depending on whether the interaction is with nuclear distribution protein nudE homolog 1 (NDE1) or p150^Glued^ (69). NDE1 protein is involved in the intracellular organization of the Golgi through interaction with nuclear distribution protein nude-like 1 (NDEL1), lyssencephaly-1 protein, and dynein; silencing of NDE1 and NDEL1 disorganizes the Golgi, makes the endocytic compartment collapse toward the plasma membrane and abrogates cortical dynein localization (70). The palmitoylation of either NDE1 or NDEL1 prevents interaction with dynein and intracellular traffic (71), thereby pointing to a relevant spatial mechanism to regulate dynein complexes composition and action. In this regard, the silencing of p150^Glued^ does not seem to exert an effect on centrosome localization at the IS in this study (69). Other authors have observed that the direct knockdown of dynein heavy chain does not affect the translocation of the centrosome in mouse cells (65). However, a number of studies support dynein/dynactin role in centrosome polarization in lymphocytes (25, 60, 69, 72, 73).

**Figure 3:**
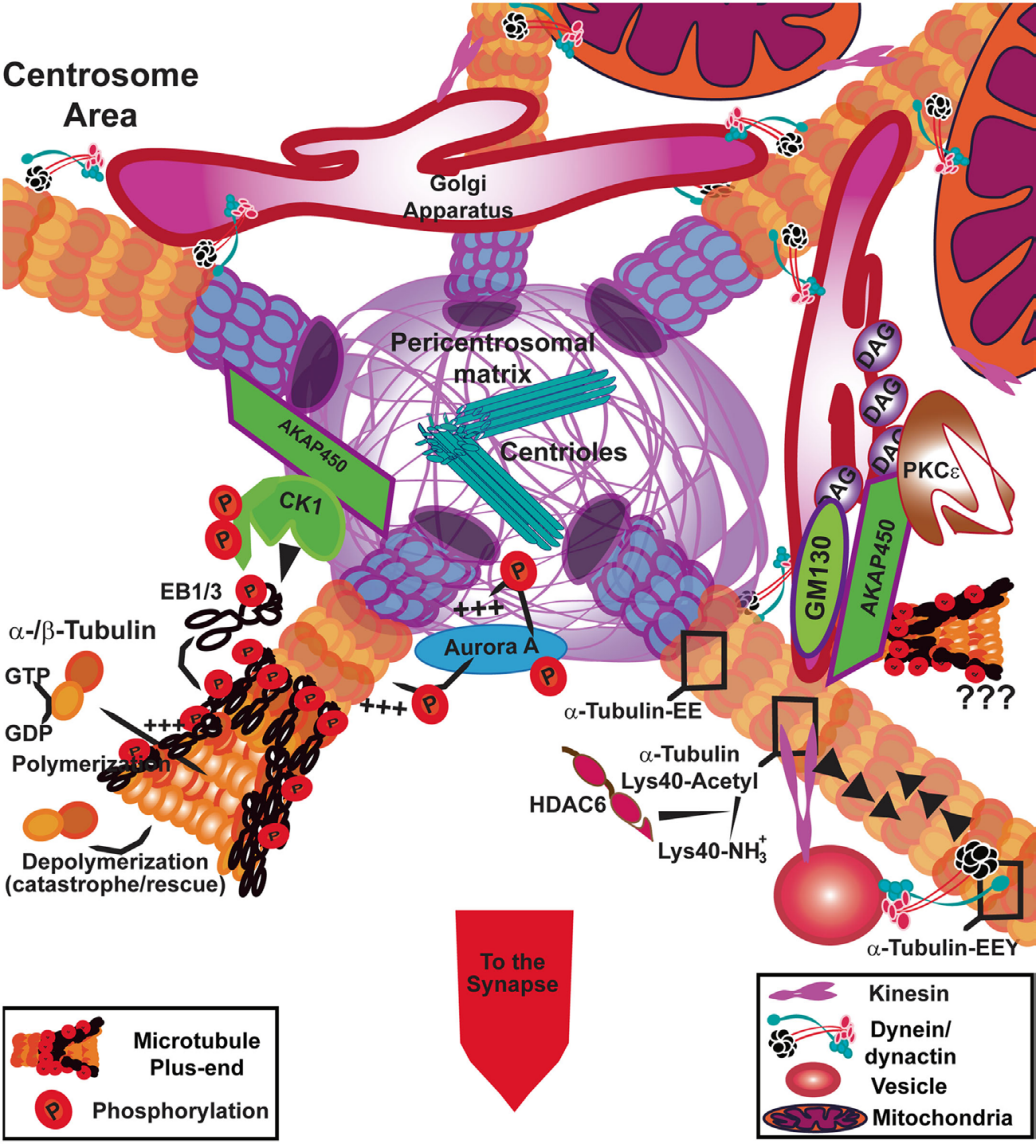
Signaling at the centrosome area to fuel tubulin polymerization. In T cells, the polymerization of microtubules (MTs) from the centrosome is controlled by casein kinase Iδ (CKIδ) through phosphorylation of end-binding 1 (EB1). AKAP450 anchors CKI to the pericentrosomal matrix. Aurora A also promotes the incorporation of α/β-tubulin heterodimers into MTs at the centrosome through its kinase activity. AKAP450 can also dock at the Golgi apparatus where it may collaborate with GM130 to facilitate tubulin polymerization. The Golgi apparatus is formed by diacylglycerol (DAG)-enriched membranes, where protein kinase C (PKC)ε anchors. AKAP450 binds to hypophosphorylated PKCε, which can constitute a reservoir for the non-activated kinase. The post-translational modifications of the MTs can affect the binding of molecular motors; kinesin likely interacts preferentially with acetylated MTs and dynein/dynactin interact with tyrosinated MT (α-tubulin-EEY) through dynactin binding, although dynein can move along detyrosinated (α-tubulin-EE) and tyrosinated MTs. Both motors are in charge of movement around the centrosomal area, of organelles such as vesicles and mitochondria, whereas dynein is the main responsible for Golgi apparatus shape and stability. The images in the figure are not scaled.

The full deletion of p150 or *Glued* is lethal early in embryo development in *D. melanogaster*. Genetic experiments to analyze the survival of deficient cells in wild-type adult tissues were unable to recapitulate the cell functionality (74). This indicates that p150^Glued^ is essential for cells to survive, divide, or participate in tissue-level organization, although dynactin complex is not required for dynein sustained motility (75). Therefore, a partial silencing of p150^Glued^ would allow the initial activity of some dynein/dynactin complexes, without major requirements for later movement and generation of pulling forces, but with a high replacement/interchange rate of dynactin between complexes. Dynein/dynactin supercomplex has a definitive different behavior in the use of different MTs as tracks, which can be due to the different post-translational modifications (PTMs) of tubulin (Figure 3); partly through the action of different carboxipeptidases at α-tubulin ends (α-Tub-EEY) (6, 76). Dynein can move on detyrosinated MTs once the movement is initiated without the participation of dynactin. The movement of centrosomes in *C. elegans* embryos depends on the interaction of dynactin with tyrosinated MTs, the cytoplasmic pulling forces exerted through its binding to dynein complex and the initiation of intracellular traffic (77). Also, dynactin interacts preferentially with tyrosinated MTs through p150^Glued^ or with the EEY-ends of end-binding (EB) proteins bound to MTs (75). The formin INF2 regulates the tyrosinated state of MTs in T cells during activation; MTs near the translocated centrosome are detyrosinated (α-Tub-EE) and TCR activation promotes the increase of detyrosinated MTs (46). A possibility is that dynactin would help dynein to initiate its processive movement to transport cargoes on tyrosinated MTs until the area of detyrosinated MTs near the centrosome is reached. Alternatively, dynactin can use EB1 or EB3 at the plus-ends of MTs. Conceivably, high inhibition of dynactin/dynein interaction by sustained overexpression of p50-dynamitin or complete knockdown of p150^Glued^ would affect dynein initial interaction with MTs, preventing intracellular traffic and localization of the centrosome at the IS and the organization of organelles due to lack of cytosolic pulling forces.

## The is and Its Axonemal Connections

The connection between IS and axonemal components is being established. Axonemal dynein is very important to allow the movement of flagella, based on its interaction with the axonemal MTs and its AAA motor activity; a “coup de force” (57, 58), which may be also a possible mechanism at the IS. During the biogenesis of the cilium, including centrosomal and acentrosomal processes, the basal body connects to cenexin–centriolin–Rab11a–Rabin8–Rab8 complex and organizes the retrograde and anterograde transport of vesicles along MTs through dynein and kinesin, respectively. These molecules also act at the IS; Rab8 and vesicle-associated membrane protein (VAMP)3 complex regulates recycling of TCRs (78). Rab8a and Rab11a dissociate from the pericentriolar region by casein kinase 1 (CK1δ) action (79). The centrosomal docking of CK1δ is mediated by AKAP450 (80) (Figure 3) and is needed to form the basal body of the primary cilium. In CD4 T cells, AKAP450 inhibition delocalizes the centrosome from the IS and decreases TCR and integrin activation and clustering (81). In other cell types, AKAP450 has an important role in MT polymerization from the centrosome and through the Golgi mediated by interaction with GM130 (82). Therefore, it can be also of relevance for cytosolic pulling forces. CK1δ silencing causes a 50% reduction in the centrosomal positioning at the IS in Jurkat cells. CK1δ forms a complex with EB1, phosphorylates it, and activates its function. It might interact with dynein/dynactin directly or through EB1, but these can represent different complexes recovered by co-immunoprecipitation and EB1-GST pulldown, respectively. CK1δ phosphorylation of EB1 can activate the protein and promote MT growing from the centrosome (Figure 3). This effect is dependent on the dynamics of CK1δ localization at the centrosome since its persistent localization at this organelle prevents the correct centrosomal polarization at the IS (83). The deletion of EB1 does not prevent centrosomal positioning at the IS, but abrogates TCR signaling at linker for activation of T cells (LAT)/phospholipase C (PLC)γ1 signalosome and regulates the traffic of CD3ζ vesicles at the IS (18). EB3, which is also expressed in T cells, could replace EB1 in allowing centrosomal positioning, since overexpression of EB1-CT as a dominant negative mutant for EB1 protein–protein interactions has an effect on centrosomal positioning (39, 84). Polymerization of MTs has been described to be important for centrosomal polarization, assayed through the use of low doses of nocodazol (25). In this study, the centrosomal relocation at the IS was defined through two different phases with different mean speeds: a first one to position the centrosome near the synapse and a second one to center and dock it. In this work, the silencing or chemical inhibition of dynein with ciliobrevin D and overexpression of a dominant negative mutant of p150^Glued^ had a negative effect on centrosomal positioning and docking (25), corroborating previous results (60). Major effects on both phases were observed upon taxol and ciliobrevin D treatment to prevent depolymerization of MTs and dynein-driven force. Taxol alone prevented partly the repositioning, as did low doses of nocodazol (loss of polymerization); therefore, a kind of internal “scanning” of the cell cortex from MT plus-ends in collaboration with dynein/dynactin was proposed as a model for docking the centrosome at the IS (25).

Acetylation, a PTM of α-tubulin, is a hallmark of stable MTs which is also detected in the cilium. HDAC6, a histone deacetylase with activity on α-tubulin or cortactin (85), is able to interact with dynein to transport unfolded proteins (86) and is also important for lymphocyte migration as a scaffold protein (87). Its role in migration in other cell types is linked to EB1 protein (88). Notably, HDAC6, which also has a role in the cilium disassembly under the control of Aurora A (89), influences CD4^+^ T cell activation at the IS, since its overexpression precludes centrosome positioning and the interaction of important signaling molecules from the TCR pathways with MTs. HDAC6-silenced CD4 T cells showed a similar hyper-acetylation of tubulin than taxol-treated cells and even higher centrosome polarization than control cells (90). Likewise, in HDAC6-deficient CD8 T cells, the polarization of the centrosome is also higher than in control cells (it is closer to the IS) and tubulin acetylation is increased (91). Taxane (paclitaxel and docetaxel) binding to MTs was mapped to the β-tubulin subunit on the MT inner surface (92); the initially accepted model proposed that taxanes and other MT-stabilizing agents reach the binding pocket by diffusing through the MT wall. However, the kinetics of binding determined that diffusion could not account for the process (93). More recently, the application of different computational techniques to the MT structure showed that a possible external binding pocket would allow an initial binding and later the entry of the drugs (94). Therefore, depending on the amount of drug present, taxol may be affecting the binding of different microtubule-associated proteins (MAPs) at both surfaces of the MT. This may account for the different consequences of taxol treatment depending on the amount of drug used (from 1 to 20 nM; minutes to hours), as known for stimulation of MT growth *in vitro* (95), formation of MT bundles due to high polymerization and stabilization (96) and cell death and mitotic inhibition (97). In the work of Yi et al., the concentration of taxane used did not seem to provoke great changes in the overall shape of MT skeleton (pre-treatment with 0.5 µM for 10 min and then, stimulation), but prevented catastrophes, and therefore stabilized MT growth. They observed a defect in both phases: repositioning and docking. The combined treatment with ciliobrevin D and taxane produced the major effect, blocking centrosome movement. Indeed, inhibition of dynein with ciliobrevin D promoted the disorganization of intracellular organelles and vesicles (25). In sum, treatment with taxanes initially promotes an ever-growing MT skeleton, depending on the dose and time of treatment, with a resulting paralyzed skeleton and high acetylation of MTs. In addition, the inhibition of molecular motors such as dynein/dynactin prevents correct organellar disposition. This may have differential consequences on the activity of molecular motors both on cytosolic and cortical pulling forces, depending on the status of the MT cytoskeleton, the organelle positioning, and the interaction with cortical surfaces.

## Motoring the Synaptic Organelles to Fuel Cytoskeletal Dynamics

Mitochondria localize at the IS (98) and this localization is also dependent on the centrosome polarization to the IS, since mitochondria are accumulated around the de-localized centrosome and perinuclear region in T cells overexpressing p50-dynamitin (72). The perinuclear localization of mitochondria upon p50-dynamitin-GFP overexpression was primarily observed in Hela cells. The recruitment of dynamin-related protein 1 (drp1), a protein involved in fission of mitochondria, to the dynactin/dynein complex was shown to sustain the retrograde transport. The size and shape of mitochondria was irregular in these cells, with some of them presenting T- and V-shapes (99). Drp1 helps the correct localization of mitochondria at the uropod, the trailing edge of migrating polarized lymphocytes, prior to stimulation to form an IS. Through mitochondria localization at the uropod, the lymphocyte regulates its ability to migrate (100). These mitochondria surround the centrosomal area, and can provide adenosine tri-phosphate (ATP) to the intracellular traffic for LFA-1 recycling and Myosin II contraction needed to sustain the motility and polarity of the lymphocyte (101). Upon TCR activation, drp1-disrupted T cells allowed the centrosomal localization during IS formation, but mitochondria were not correctly polarized to the IS. There was no effective movement of the mitochondria toward the minus-end of MTs. The required ATP production for energy fueling at the IS was also diminished (72). The effect of drp1 delocalization from mitochondria upon p50-overexpression may also be due to a defective fission of mitochondria, which are then poorly transported in an anterograde mode by kinesin-1.

Kinesin-1, also called conventional kinesin, is a MT plus-end-oriented motor complex with different subunits. Kinesin-1 is a heterotetramer of ~380 kDa and comprises two kinesin heavy chains (KHCs) with motor activity and two kinesin light chains (102). It is important to note that kinesins are an extended family of proteins, with about 45 genes coding for them. Most of them [kinesin family members (KIFs)] have their motor domain at the N-terminus, but also as a central domain or at the C-terminus, determining whether they walk toward the plus-ends (N-KIF) or the minus-end (C-KIF) of the MT (103). Kinesin-1, an N-KIF, uses adaptor proteins to fix the cargo; for mitochondria effective movement forward to the cell cortex (MT plus-ends), Miro-1 forms a triade with Milton and kinesin-1 (104). The role of Miro-1 protein has been reported in the localization of mitochondria during T cell–endothelial contact for transmigration from blood vessels to tissues during inflammation. Miro-1 is needed to relocate the mitochondria around the centrosome, which is recruited from the uropod (trailing edge) to the T cell–endothelial cell contact area and congregates the mitochondria there (73). Miro-1 interacts with the dynactin subunit p150^Glued^ and dynein heavy chain in these lymphocytes, but the possible complex formed with kinesin-1 was not explored. Therefore, motors might coordinately interchange at the surface of the cargoes, to regulate the retrograde and anterograde transport through MT, using the centrosome as a crossroads.

Kinesin-1 is indeed involved in the final transport and delivery of lytic granules at the killing IS in CTLs, forming a complex with Slp3 and Rab27a (105). In fact, the knockout mouse for *Kif5b*, the KHC involved, is embryonic lethal, showing perinuclear clustering of lysosomes and mitochondria. Kinesin-1 is helped in the transport of lytic granules to the IS by the action of HDAC6 (91). In HDAC6 knockout T cells, the acetylation of MTs is highly increased, but the centrosomal polarization to the IS of either silenced CD4 or knockout CD8 T cells was even enhanced (90, 91). Kinesin-1 ability to bind and move over MT is increased by acetylation at Lys40 of α-tubulin in the lumen of MTs (106). This is in concert with the long-term increase in acetylated MTs at the IS (90) and would facilitate the kinesin-driven movement of vesicles from centrosomal region to the plasma membrane at the IS. Indeed, the use of cell-extracts of intact MT networks and single fluorescently labeled motor proteins to study motility through total internal reflection fluorescence microscopy (TIRFm) unveiled that acetylated MTs are predominantly bundled, which enhances the number of kinesin binding sites and run lengths of the motor (107). However, in the case of HDAC6 knockout CD8 T cells, the kinesin-1 interaction with p150^Glued^ was defective, correlating with a defect in the final delivery of lytic granules at the IS and their degranulation, even though acetylation of MTs was highly increased (91).

Histone deacetylase 6 may also play a role in the biogenesis and degradation of organelles through interaction with dynein/dynactin. These proteins are well-known partners for the transport of ubiquitinated, misfolded proteins to the aggresome formed near the centrosome for degradation through autophagy. Its interaction with dynein/dynactin takes part through a region different from its two catalytic domains for acetylation and its C-terminus (86). Parkin coordinates the E2 enzyme UbcH13/Uev1a to mediate K63-linked polyubiquitination of misfolded proteins (108). Under conditions of proteasomal impairment, the machinery and membranes for autophagosome are recruited to the aggresome and the fusion with lysosomes allows protein clearance. Indeed, the recruitment of Parkin to the centrosome in these conditions is dependent on HDAC6. This accumulation was reversible and HDAC6 used either dynein or kinesin-1 for bidirectional movements (109). HDAC6 binds preferentially to K63-ubiquitin modified proteins (instead of K48) through its ubiquitin-binding domain, at the C-terminal (110). It may bind both mono or polyubiquitin chains (111, 112), although it seems that it prefers polyubiquitinated chains (108). This precise relationship between HDAC6 and the dynein motor is likely to be of relevance for mitochondrial shape and health, since HDAC6 and Parkin are both involved in the process of mitophagy (56). Therefore, the possible connections in the cytosolic and cortex pulling forces generated by dynein and their relationship with kinesin complexes and their ability to interconnect organelles and to move components inside the cell is a field for intense research. In this context, the MTs, their PTMs, and enzymatic modifiers will be extremely relevant.

Myosins are a superfamily of motor proteins that bind to actin and use the energy of ATP hydrolysis to generate force and movement along actin filaments. There are about 18 classes of myosins. They play significant roles in cell movement, muscle contraction, cytokinesis, membrane trafficking, and signal transduction (113). They consist of a motor domain, a neck region, and a tail region; most myosins form a dimer of two heavy chains with the supplementary binding of two light chains (MLC) per heavy chain to their neck region. Regulatory MLCs can be phosphorylated for regulation of the motor activity (103). Non-muscle myosin IIA (encoded by gene *Myh9*) has been involved in the accumulation of the TCR to the center of the IS to be recycled (114). The lack of mitochondria polarization at the IS by inhibition of drp1 prevented MLC phosphorylation at Ser19 at the actin-rich lamella, thereby unleashing TCRs from the retrograde flow, which showed a less concentrated appearance. However, the centrosome was correctly positioned at the IS (72). The collaboration between myosin IIA and dynein has been recently shown in mouse cells through the use of photoactivatable peptide-MHC complexes. This evidence supports the action of Myosin IIA in pushing the centrosome toward the IS while dynein would pull it from the IS. The inhibition of each one separately did not exert apparent high effects on centrosomal positioning in this study. Indeed, inhibition of Myosin IIA did not alter the signaling from the TCR (115). Dynein localization at the plasma membrane has been suggested to precede the centrosome polarization at the IS rapidly upon TCR activation. The gradient of diacylglycerol (DAG) organized at the IS by active PLCγ1 would be the polarizing signal to direct centrosomal localization (116). In this sense, the MAP4 knockdown reduces the stability of MTs and makes the centrosome to move slower until it reaches the IS, although PLCγ1 is more active and DAG accumulation increases at the IS. It is feasible that centrosomal polarization acts as a negative regulator for DAG production. DAG accumulation at the IS is also observed when Ct-AKAP450-GFP is overexpressed in T cells (117), a construct that displaces AKAP450 from the centrosome and prevents its translocation to the IS (81). Indeed, if DAG production is disturbed, the centrosome does not position correctly (116). To control centrosomal positioning, there is a specific and temporal recruitment of three different protein kinases C (PKCs) to the IS; essentially PKCε and PKCη come first followed by PKCθ (118). All of them bind to DAG and phorbol esters and need phosphorylation by 3′-phoshoinositide-dependent protein kinase 1 at their activation loop to be fully active (119). PKCε controls its localization through second messengers and is dependent on G-protein-coupled receptors activation. It can bind to Myosin IIA and actin in fibroblasts, thereby connecting to actin cytoskeleton (120). In this regard, the exposure to the CXCL12 chemokine strengthens the IS shape, as an additive signal to CD3 and CD28 (121), and CXCR4, the G-protein-coupled receptor for CXCL12, is localized at the IS through connection with actin cytoskeleton (122). CXCL12 binding to its receptor allows Ca^2+^ influx and rapid activation of Rac1 (123) and its internalization seems dependent on MIIA interaction (124). Immature hypophosphorylated PKCε associates to AKAP450 (125) and this can be related to its high basal localization to DAG-enriched membranes, such as the Golgi (126) (Figure 3). PKCθ is a well-known modulated kinase during T cell activation, which recruitment to the IS depends on CD28 costimulation (127). PKCθ clustering at the center of the IS correlates with its activity (127) and is highly dependent on actin dynamics; Golgi-resident eNOS translocated to the IS together with the centrosome lowers the actin retrograde flux and enhances PKCθ activity (32). The regulation of PKCθ downstream activity by the control of Carma1 localization at the IS by the plus-end directed, kinesin molecular motor GAKIN has also been described. GAKIN can walk on the MTs toward the periphery of the IS, displacing Carma1 from Bcl10 and the central part of the IS (128). Recently, the identification of a protein complex comprising CD28, Lck, and PKCθ has explained the dependence of PKCθ on CD28 activation. The unique domain V3 from PKCθ interacts with the SH3 domain of Lck, which in turn docks at the phosphorylated tail of CD28 (129). The mutation of the PI3K interaction site in CD28 prevented the recruitment of PKCθ at the IS and transcription of IL-2 mRNA (130), determining the relevance of the targeting to the IS in T cells. The localization through Lck at the IS may explain also why PKCθ docks at the IS in CD28-deficient Jurkat T cells (32). A different mechanism seems to operate in regulatory T cells (Tregs), with PKCθ preferentially located at the distal pole of the T cell, far from the T-APC contact (131). The use of knockout mice or chemical inhibitors for PKCθ has rendered distinct results in the Treg subset. Hence, Tregs from *Pkc*θ^−^*^/^*^−^ showed similar activity than wild type, although their numbers were diminished due to developmental problems (132), while chemical inhibition clearly enhanced Treg function (131). IL-2 production by effector T cells is essential for CD4 Treg differentiation and function (133), which could affect the numbers in the *Pkc*θ^−^*^/^*^−^ mice. Naïve T cells from *Pkc*θ^−^*^/^*^−^ mice have been analyzed for stability of the IS, and the absence of the kinase allows the IS to be formed for longer periods of time without loss of symmetry, therefore preventing the formation of different IS by the same T cell (134). Therefore, the interconnection between skeletons and signaling is clear again, but there is still much information lacking to understand precisely which is the signaling cascades controlling the motor activities and the dynamics of the actin-based and tubulin-based skeletons at the IS.

## Protein Multiplexing Highlights the Complexity of Transport Systems

The use of different imaging techniques together with biochemical identification of proteins and different drugs against cytoskeletal components has allowed the understanding of different routes for transport of vesicles at the IS. The intracellular traffic at the IS has been analyzed mainly through wide-field fluorescence microscopy, laser scanning confocal microcopy (LSCM), and, to a lesser extent, TIRFm (48, 135). Endocytosed TCRs enter a pathway to recycling endosomes marked by Rab4 and Rab11. Rab4-positive endosomes are early endosomes involved in rapid shuttling of internalized receptors to the plasma membrane in an MT-independent manner. The endosomes marked by Rab11 cluster deeper inside the cell (next to the centrosome) and follow a slower route to return to the plasma membrane along MT. Rab35 and other Rab GTPases regulate the endosomal trafficking together with Wiskott–Aldrich syndrome protein and SCAR homolog (WASH), which controls actin polymerization. WASH activates Arp2/3 complex and also interacts with tubulin cytoskeleton in both early and late endosomes, promoting the local actin polymerization that may provide the force for their movement along MT (78). Later, actin clearance at specific sites of the IS allows the fusion of the vesicles with the plasma membrane. The corresponding N-ethylmaleimide-sensitive factor attachment protein receptors (SNAREs) at the vesicles (v-SNARE) and on target membranes (t-SNAREs) mediate this process. A complex between two t-SNAREs (syntaxin-3 or -4 and SNAP-23 in non-neuronal cells) and one v-SNARE such as VAMP3 (136) allows the docking and priming of the vesicle, which fuses with the plasma membrane in the presence of high Ca^2+^. In contrast to TCR/CD3 vesicles that are controlled by VAMP3, the endosomal recruitment and docking of LAT to the cortical region of the IS are dependent on the VAMP7 v-SNARE (although CD3 vesicles may also interact with VAMP7) (137). The presence of two traveling LAT pools at the IS was described through fluorescence microscopy (138). Endosomal pools of LAT are localized in different subpopulations of recycling endosomes marked by Rab27 and Rab37. This suggests that early phosphorylation of LAT upon TCR activation depends on the clustering of the LAT pool at the plasma membrane rather than on the LAT subset at endosomes. The latter seems to be more involved, however, in stabilizing signaling mediators close to the TCR (19, 139). The growing of MTs at the IS, analyzed by TIRFm through imaging of EB1 and EB3 (18), allows the movement of TCR/CD3-enriched cortical vesicles underneath the plasma membrane and their encounter with LAT-enriched vesicles, thereby helping sustained activation of LAT and PLCγ1 upon TCR triggering (18). Indeed, an important pool of Lck in Rab11b^+^MAL^+^ endosomes is detected during T cell activation. MAL is involved in the targeting of Lck to the plasma membrane and the correct sorting of Lck and LAT to membrane subdomains at the IS (84, 140). Rab11b interacts with myosin 5B, a motor protein able to interact with actin cables and vesicles, through the adaptor protein uncoordinated 119 (Unc 119). This complex allows the final delivery of the vesicles, from the MTs of the pericentrosomal region to the cortical actin at the IS (4). This kind of collaboration between motors and skeletons for intracellular traffic is essential for correct T cell activation, and probably favors cytosolic pulling forces.

An unexpected player was recently described as a regulator of TCR recycling at the IS: the intraflagellar transport (IFT) system. IFT are multimeric protein complexes relevant for the biogenesis and maintenance of the primary cilium. T cells express different IFT constituents such as IFT20, 52, 57, and 88, which participate in the recycling of the TCR to the endosomal system upon centrosome positioning at the IS. The polarization of the Golgi apparatus and the centrosome drives the building of both structures, the IS and the cilium. They direct the growing of MT and the traffic of vesicles toward the plasma membrane. These membranes are both highly enriched in cholesterol and sphingolipids. They act as signaling platforms for extracellular cues (21). IFT20, together with IFT88, IFT52 and IFT57, are recruited to the IS in association with the Golgi apparatus and centrioles (141). IFT20 sustains TCR clustering and signaling but is dispensable for polarization of the Golgi and the centrosome. IFT20 couples internalized TCR/CD3 complexes with Rab5^+^ early endosomes and promotes their transit to recycling endosomes. Since IFT20 co-localizes with the TCR in Rab11^+^Rab4^+^ endosomes, it is possible that it accompanies this receptor during recycling. In this regard, IFT20 also interacts with the transferrin receptor (TfR), which also undergoes polarized recycling at the IS (141, 142). Indeed, tubulin heterodimers can be transported by the IFT system to the end of the cilium, thereby facilitating its elongation (143). An increase of available heterodimers helps reaching the critical concentration needed for polymerization of MTs. A similar process may take place at the IS, providing the IFT proteins expressed by the T cell can also perform this task. Therefore, the transport of molecules and vesicles and its relationship with the centrosome and the MTs arising from it seem to be of special relevance in different cell systems used for sensing changes in the extracellular medium, such as the cilium and the IS. Intracellular traffic and the cytoskeleton are tightly related in the regulation of the IS and the cilium. Tubulin tracks, as identified by cryo-tomography or transmission electron microscopy near mitochondria and the endoplasmic reticulum (24, 66, 72) connect different organelles, and establish tensional forces between them, as well as with the plasma membrane. Pulling and pushing forces would help the scission and fusion of vesicles from and to Golgi or endosomes, respectively. For instance, EB1, which is involved in MT growth from the polarized centrosome and regulation of vesicular traffic in T cells, is also related to the vesicular transport for cilium formation (144). Indeed, during cilium formation, the tubule scission from the Golgi by spastin, an MT-severing protein, is achieved through the interaction with the ESCRT complex (145). Therefore, the linkage between intracellular organelles and cytoskeleton is needed to organize a productive IS, and this is in part mediated by different molecular complexes that coordinate their action.

## Concluding Remarks

Signaling constituents transported to the IS by polarized vesicles trafficking are a crucial piece of the information transmitted from the plasma membrane to the nucleus and other organelles (18, 19, 146). This is needed to sustain T cell activation and is activated from the TCR, costimulatory molecules such as CD28 and adhesive receptors such as LFA-1. The different cytoskeletal systems are absolutely required to coordinate and activate the plethora of molecules involved in these processes. Molecular motors facilitate these events by exerting forces of different orientations along the cytoskeletal track. These pulling and pushing forces are critical for cell shaping and movement. Future experimentation will profit from new technical advances to analyze complete cells in three dimensions with higher resolution and low toxicity for live cells such as LLSM or 3D-SIM. The super-resolution techniques such as STED and photo activated localization microscopy (PALM) or stochastic optical reconstruction microscopy (STORM) that have been developed will allow to analyze in more detail the already known structures [for revision of imaging techniques, see in this topic (147) and for protocols and methods (148)]. To unveil the role of motors in T cell organization, it is essential to study the specific composition of the complexes they form, the organelles they bind to and their relationship with the dynamics of the cytoskeleton systems, in particular with the PTMs that can fine-tune their functional activity and direct the activation of T cells. Dynein/dynactin is a crucial motor complex in this context. It directs the rearrangement of different MT-associated organelles, such as the Golgi and mitochondria. The sum of the forces exerted from the cell cortex and the cytosolic elements will determine the shape of the IS.

## Author Contributions

NM-C wrote the manuscript and composed the figures. FS-M wrote the manuscript.

## Conflict of Interest Statement

The authors declare that the research was conducted in the absence of any commercial or financial relationships that could be construed as a potential conflict of interest.

## Abbreviations

ADAP/SLAP130/FYB, adhesion and degranulation promoting adaptor protein/SLP-76-associated phosphoprotein/fyn-binding protein; AKAP450, A kinase-anchoring protein of 450 kDa; APC, antigen-presenting cell; ATP, adenosine tri-phosphate; CD, cluster of differentiation; CK, casein kinase; CTL, cytotoxic T-lymphocyte; CTLA, cytotoxic T-lymphocyte-associated antigen; DAG, diacylglycerol; DC, dendritic cell; EB1, EB3, end-binding 1 or 3; eNOS, endothelial nitric oxide synthase, FH, formin homology; FIP, arfophilin; FMNL, formin-like protein; ICAM, intercellular adhesion molecule; IFT, intraflagellar transport; INF2, inverted formin-2; IS, immune synapse; HDAC6, histone deacetylase 6; KIF, kinesin family member; KLC, kinesin light chain; KHC, kinesin heavy chain; LAT, linker for activation of T cells; Lck, leukocyte C-terminal Src kinase; LFA-1, lymphoctye function-associated antigen-1; LIS1, lyssencephaly-1 protein; LLSM, lattice light-sheet microscopy; LSCM, laser scanning confocal microscopy; MAP, microtubule-associated protein; mDia, mammal diaphanous-related-formin; MHC, major histocompatibility complex; MT, microtubule; NDE1, nuclear distribution protein nudE homolog 1; NDEL1, nuclear distribution protein nude-like 1; NO, nitric oxide; PALM, photo activated localization microscopy; PDK, 3′-phoshoinositide-dependent protein kinase; PKC, protein kinase C; PLC, phospholipase C; Rac, ras-related C3 botulinum toxin substrate-1; SKAP55, Src kinase-associated phosphoprotein; SNARE, N-ethylmaleimide-sensitive factor attachment protein receptors; STED, stimulated emission depletion; STORM, stochastic optical reconstruction microscopy; TCR, T cell receptor; TIRFm, total internal reflection fluorescence microscopy; VAMP, vesicle-associated membrane protein.
